# Image-Based Identification and Genomic Analysis of Single Circulating Tumor Cells in High Grade Serous Ovarian Cancer Patients

**DOI:** 10.3390/cancers13153748

**Published:** 2021-07-26

**Authors:** Carolin Salmon, Janina Levermann, Rui P. L. Neves, Sven-Thorsten Liffers, Jan Dominik Kuhlmann, Paul Buderath, Rainer Kimmig, Sabine Kasimir-Bauer

**Affiliations:** 1 Department of Gynecology and Obstetrics, University Hospital Essen, 45147 Essen, Germany; Janina.Levermann@gmx.de (J.L.); Paul.Buderath@uk-essen.de (P.B.); Rainer.Kimmig@uk-essen.de (R.K.); Sabine.Kasimir-Bauer@uk-essen.de (S.K.-B.); 2 Department of General, Visceral and Pediatric Surgery, University Hospital and Medical Faculty of the Heinrich-Heine University Düsseldorf, 40225 Düsseldorf, Germany; Rui.Neves@med.uni-duesseldorf.de; 3 West German Cancer Center, Bridge Institute of Experimental Tumor Therapy, University Medicine Essen, 45147 Essen, Germany; Sven-Thorsten.Liffers@uk-essen.de; 4 German Cancer Research Center (DKFZ) and German Cancer Consortium (DKTK), Partner Site Essen, Division of Solid Tumor Translational Oncology, 69120 Heidelberg, Germany; 5 Department of Gynecology and Obstetrics, Medical Faculty and University Hospital Carl Gustav Carus, Technische Universität Dresden, 01307 Dresden, Germany; Jan.Kuhlmann@uniklinikum-dresden.de; 6 German Cancer Consortium (DKTK), Partner Site Dresden and German Cancer Research Center (DKFZ), 69120 Heidelberg, Germany; 7 National Center for Tumor Diseases (NCT), 01307 Dresden, Germany; 8 German Cancer Research Center (DKFZ), 69120 Heidelberg, Germany; 9 Faculty of Medicine and University Hospital Carl Gustav Carus, Technische Universität Dresden, 01307 Dresden, Germany; 10 Helmholtz-Zentrum Dresden—Rossendorf (HZDR), 01307 Dresden, Germany

**Keywords:** single circulating tumor cell, liquid biopsy, ovarian cancer, copy number variations

## Abstract

**Simple Summary:**

Ovarian Cancer (OC) is one of the leading causes of death among gynecological tumors and there is still an insufficient understanding of its evolution. Blood, as a minimal invasive tool, allows multiple sampling over the treatment course and genomic single circulating tumor cell (sCTC) data provide the opportunity to investigate the genetic tumor evolution. CTC detection in OC remains difficult, due to epithelial-mesenchymal transition (EMT). This proof of principle study presents a workflow to generate sCTC genomic data, with the need of further studies to improve the CTC detection rate and enable insights into tumor evolution on a sCTC resolution to identify new treatment targets and/or biomarkers for an early treatment intervention.

**Abstract:**

In Ovarian Cancer (OC), the analysis of single circulating tumor cells (sCTCs) might help to investigate genetic tumor evolution during the course of treatment. Since common CTC identification features failed to reliably detect CTCs in OC, we here present a workflow for their detection and genomic analysis. Blood of 13 high-grade serous primary OC patients was analyzed, using negative immunomagnetic enrichment, followed by immunofluorescence staining and imaging for Hoechst, ERCC1, CD45, CD11b and cytokeratin (CK) and sCTC sorting with the DEPArray^TM^ NxT. The whole genome of single cells was amplified and profiled for copy number variation (CNV). We detected: Type A-cells, epithelial (Hoechst^pos^, ERCC1^pos^, CD45^neg^, CD11b^pos^, CK^pos^); Type B-cells, potentially epithelial (Hoechst^pos^, ERCC1^pos^, CD45^neg^, CD11b^pos^, CK^neg^) and Type C-cells, potentially mesenchymal (Hoechst^pos^, ERCC1^pos^, CD45^neg^, CD11b^neg^, CK^neg^). In total, we identified five (38.5%) patients harboring sCTCs with an altered CN profile, which were mainly Type A-cells (80%). In addition to inter-and intra-patient genomic heterogeneity, high numbers of Type B- and C-cells were identified in every patient with their aberrant character only confirmed in 6.25% and 4.76% of cases. Further identification markers and studies in the course of treatment are under way to expand sCTC analysis for the identification of tumor evolution in OC.

## 1. Introduction

Ovarian Cancer (OC) is one of the leading causes of death among gynecological tumors, because of late-stage diagnosis and frequently occurring disease relapses. High-grade serous ovarian cancer (HGSOC) is the most frequent histological subtype and late stage diagnosis is strongly correlated with worse prognosis [1]. Despite studies investigating primary OC tumor tissue, identifying common TP53 mutation and recurrent BRCA, NF1, RB1 and CDK12 mutations [2], OC is still not well understood. Most cancers can be linked to driver mutations, causing disease progression, whereas OC is commonly characterized by a chromosomal instability (CIN), resulting in great amounts of copy number alterations (CNA) with TP53 mutations being only one of the genetic driving forces [2,3,4]. Currently, novel surface antigen expressions such as CD11b, unexpectedly expressed in OC tissues, are expanding the knowledge of potential actionable targets in OC [5]. However, there is still an insufficient understanding of OC disease evolution, since tumor tissue is usually only available at primary diagnosis.

To circumvent this problem, blood, as a minimally invasive tool, has frequently been used because it allows multiple sampling over the treatment course for the detection and characterization of different analytes. In this context, the characterization of circulating tumor cells (CTCs) has the great potential to provide insights into the driving components of disease progression and poor treatment response in OC patients. The prognostic value of CTCs with regard to progression free survival (PFS) and overall survival (OS) has already been demonstrated [6,7]. CTC characterization at primary diagnosis revealed that the presence of excision repair cross-complementing group 1 (ERCC1)- positive CTCs, an endonuclease associated with DNA repair, was an independent predictor for clinical platinum-resistance, whereas ERCC1-expression in corresponding primary tumor tissue predicted neither platinum-resistance nor prognosis [8]. Furthermore, auxiliary assessment of ERCC1-transcripts expanded the phenotypic spectrum of CTC-detection and defined an additional fraction of CTCs [9]. Comparable data were also demonstrated by Obermayr et al., who identified a subfraction of CTCs, over-expressing the peptidylprolyl isomerase C (PPIC) gene, which correlated with poor patient outcome, independently of classical clinicopathological parameters [7].

Unfortunately, common CTC identification features, mostly epithelial characteristics such as EpCAM and Cytokeratin (CK), successfully used for CTC-analysis in a variety of solid tumors [10], failed to reliably detect CTCs in OC [11,12]. Epithelial- mesenchymal plasticity (EMP) is a common ability of tumor cells entering the bloodstream to provide fitness and flexibility in different environments [13]. The co-existence of epithelial- and mesenchymal features in CTCs has already been demonstrated in OC [14], explaining the limits of common CTC detection methods. Many other studies have investigated the transcriptional programs of CTCs in OC by analyzing enriched CTC fractions, either using epithelial and mesenchymal [15,16] or label-free enrichment methods [7,17,18]. Most of the applied technologies were used to investigate CTC enriched fractions, only providing limited information on CTC heterogeneity. In this regard, genomic single CTC (sCTC) characterization [19], successfully applied in other cancer entities [20], could provide the opportunity of investigating the genetic tumor evolution in OC. This will not only be crucial for a better understanding of OC, but will also enable one to dissect genetic CTC heterogeneity on a single cell resolution to identify new treatment targets and/or biomarkers for an early treatment intervention.

We here present a newly established workflow for sCTC detection and characterization in blood samples of 13 HGSOC patients at primary diagnosis, applying MACS technology for negative immunomagnetic enrichment with CD45 and CD235a antibodies, followed by sCTC sorting using the DEPArray^TM^ NXT based on fluorescence imaging for Hoechst, ERCC1, CD11b, CK and CD45 and subsequent whole genome amplification (WGA) to determine single cell copy number variation profiles.

## 2. Materials and Methods

**Figure 1:**
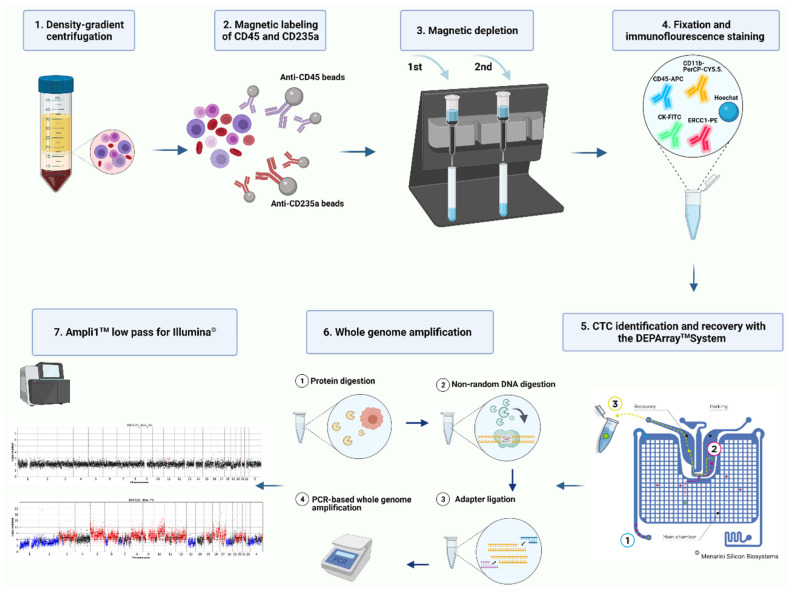
Workflow for single CTC isolation and molecular characterization in blood samples of HGSOC patients. After density-gradient centrifugation and magnetic-based negative depletion of erythrocytes (CD235a) and leucocytes (CD45), immunofluorescence staining was performed. Subsequent single cell imaging and sorting using the DEPArray^TM^ Nxt was followed by a whole genome amplification. After Ampli1^TM^ low pass (Menarini Silicon Biosystems) library preparation, copy number variation sequencing was performed. Figure was created with BioRender.com.

The established workflow is illustrated in Figure 1.

### 2.1. Cell Culture and Spike-In Experiments

The human OC cell line (OVCAR-3) and the human breast cancer cell lines (SK-BR-3 and MCF-7) were purchased from American Type Culture Collection (ATCC©, Manassas, VS, USA). OVCAR-3 cells were cultured in RPMI 1640 (Gibco, Life Technologies Limited, Paisley, UK), SK-BR-3 cells in McCoy’s 5a (Gibco, Life Technologies Limited) and MCF-7 cells in MEM (Gibco, Life Technologies Limited,), containing 10% fetal bovine serum (Sigma-Aldrich Chemie GmbH, Steinheim, Germany), 1% Penicillin—Streptomycin (10,000 U/mL, Sigma-Aldrich Chemie GmbH) and 1% (0.76% in MCF-7 cells) L-Glutamin (200 mM, Gibco, Life Technologies Limited). 2.9% sodium bicarbonate (7.5% solution, Gibco, Life Technologies Limited) was added to the culturing mix of SK-BR-3 cells, whereas 2% sodium bicarbonate (7.5% solution, Gibco, Life Technologies Limited), 1% MEM non-essential amino acids solution (100X) (Gibco, Life Technologies Limited), 1% sodium pyruvate (100 mM, Gibco, Life Technologies Limited) and 0.06% human recombinant Insulin (4 mg/mL, Gibco, Life Technologies Limited) was added to the MCF-7 culturing media. All cells were cultured at 37 °C with 5% CO2 and sub-culturing was performed twice a week.

**Figure A1:**
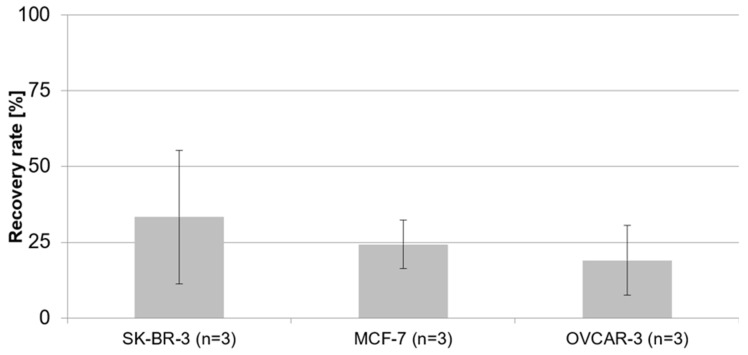
Recovery rates of different cell lines spiked into 10 mL of healthy donor blood, undergoing the enrichment procedure followed by single cell sorting using the DEPArray^TM^. For each sample, an approximate number of 40,000 cells was investigated (sample size was set by the loading capacity of one DEPArray^TM^ cartridge), the Hoechst^pos^ and CK^pos^ detected cells were then extrapolated to the actual sample size.

**Figure A2:**
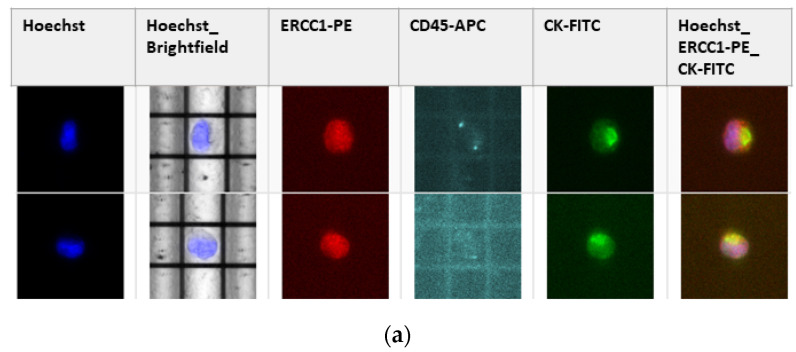

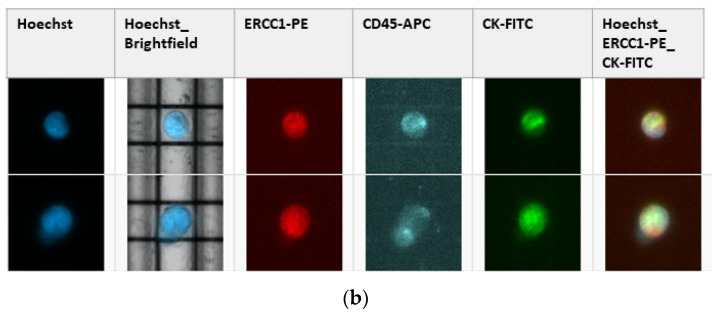
Final ERCC1- staining applied in two different healthy donor (hd) samples with spiked OVCAR-3 cells. Fluorescence panel generated with the DEPArray™ NxT. (**a**) Two OVCAR-3 cells Hoechst^pos^, ERCC1^pos^, CD45^neg^, CK^pos^ in hd1. (**b**) Two OVCAR-3 cells Hoechst^pos^, ERCC1^pos^, CD45^neg^, CK^pos^ in hd2.

**Figure A3:**
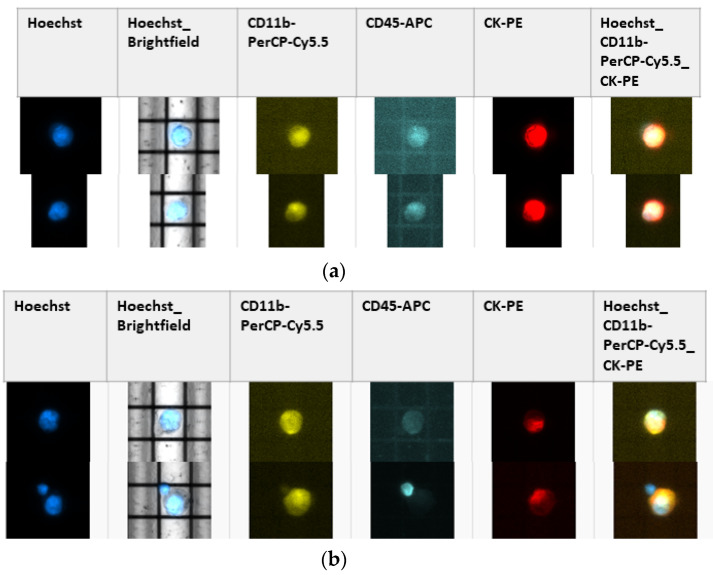
Final CD11b- staining applied in two different healthy donor (hd) samples with spiked OVCAR-3 cells. Fluorescence panel generated with the DEPArray™ NxT. (**a**) Two OVCAR-3 cells Hoechst^pos^, CD11b^pos^, CD45^neg^, CK^pos^ in hd3. (**b**) Two OVCAR-3 cells Hoechst^pos^, CD11b^pos^, CD45^neg^, CK^pos^ in hd4, the latter cell is accompanied by a CD45^pos^ leucocyte.

To test the CTC enrichment procedure in all three cell lines, an approximate number of 50 cells was added to 10 mL Ethylenediaminetetraacetic acid (EDTA) blood from healthy donors and recovery rates were estimated (Appendix A, Figure A1). To optimize ERCC1- and CD11b- antibody concentration and DEPArray^TM^ NxT settings for single cell detection (Appendix A, Figure A2 and Figure A3), as described below, an approximate number of 1000 OVCAR-3 cells were added to 10 mL EDTA blood from healthy donors.

### 2.2. Characterization of Study Patients

The present study was conducted at the Department of Gynecology and Obstetrics at the University Hospital of Essen and Dresden, Germany. In this case, 13 high-grade serous ovarian cancer patient samples were collected at the time of primary diagnosis between January and September 2020. Patient characteristics are summarized in Table 1. Informed and written consent was obtained from all patients, the study was approved by the Local Ethic Committees (Essen: 17-7859-BO; Dresden: EK236082012) and performed according to the declaration of Helsinki.

### 2.3. CTC Enrichment

10 mL EDTA blood from each patient was collected and diluted with 20 mL PBS pH 7.2 (Gibco, Life Technologies Limited)/2 mM EDTA (Invitrogen by Life Technologies, New York, NY, U.S.A.), followed by density-gradient centrifugation (10 min at 1000× *g*) using Ficoll-Paque^TM^ (GE-healthcare, Uppsala, Sweden) in Leucosep^TM^ Tubes (Greiner bio-one GmbH, Frickenhausen, Germany). The mononuclear cell layer was collected and washed with PBS (Gibco, Life Technologies Limited) and cells were counted and incubated with MicroBeads (20 µL beads per 10^7^ cells) targeting Glycophorin-A (CD235a) (human, 130-0550-501, Miltenyi Biotec, Bergisch Gladbach, Germany) and CD45 (human, 130-045-801, Miltenyi Biotec) and separated twice on LS Columns (130-042-401, Miltenyi Biotec) according to the manufacturer‘s protocol. The resulting cell suspension was fixed with paraformaldehyde (2% PFA, Sigma-Aldrich Chemie GmbH, Steinheim, Germany, for 20 min at RT) and blocked with bovine serum albumin (3% BSA, Sigma-Aldrich Chemie GmbH, for 10 min at RT) according to the immunofluorescence protocol from Menarini Silicon Biosystems.

### 2.4. Immunofluorescence Staining and Single Cell Recovery with DEPArray™ NxT

All antibodies were filtered with Ultrafree^®^-MC sterile centrifugal units (0.65 µm, Merck Millipore Ltd., Tullagreen, Ireland) before use. Fixed samples were first stained with anti-CD11b coupled to PerCp-Cy5.5 (clone CBRM1/5, sc-23934 PCPC5, Santa Cruz Biotechnology Inc., Dallas, TX, USA) for 20 min at 2–8 °C, dilution 1:4 and with anti-CD45 coupled to APC (clone 5B1, 130-113-114, Miltenyi Biotec) simultaneously for the last 10 min at 2–8 °C, dilution 1:10, in autoMACS Running Buffer (Milenyi Biotec). After a washing step with 1 mL of autoMACS Running Buffer (Milenyi Biotec) and centrifugation for 10 min at 400× *g*, permeabilization with 0.1% TitonX (Roth, Karlsruhe, Germany) diluted in autoMACS Running Buffer (Milenyi Biotec) was performed for 20 min at RT. After another washing-step, anti-ERCC1 coupled to PE (clone D-10, sc-17809, Santa Cruz Biotechnology Inc.), dilution 1:17 and anti-CK coupled to Alexa Fluor^®^ 488 (C-11, ab187773, Abcam, Cambridge, UK), dilution 1:9, were added for 20 min at 2–8 °C for intracellular staining. Following one further washing step, Hoechst 33342 (Sigma-Aldrich Chemie GmbH, dilution 0.001 mg/mL, for 5 min at RT) was added to visualize the cell nucleus. After final washing, the cell suspension was stored in the dark at 2–8 °C.

Stained cell suspensions were analyzed within a maximum of 4 days, using the DEPArray™ NxT (Menarini Silicon Biosystems, Bologna, Italy) and single cells were selected manually based on fluorescence labeling and morphology. Selected single cells were recovered and volume reduction was performed according to the instruction manual from Menarini Silicon Biosystems and isolated cells were stored at −80 °C until later downstream analyses.

### 2.5. Whole Genome Amplification and Molecular Characterization of Isolated Single Cells

The genetic material of the isolated single cells (representative for each cell population in every patient) was amplified using the adapter–linker PCR, based on MseI digestion as described [21,22] which is now commercially available as the Ampli1^TM^ whole genome amplification (WGA) kit by Menarini Silicon Biosystems. The genomic integrity index (GII) was used to assess the quality of the WGA [23] (Ampli1™ QC Kit, Menarini Silicon Biosystems). WGA was performed with 111 samples, and 77 (69.4%) presented quality compatible with further downstream analysis of copy number variation (CNV) using the low pass next-generation sequencing [24]. Ampli1™ low pass library preparation was performed by Menarini Silicon Biosystems using the Hamilton Microlab STARlet platform (Hamilton company, Reno, NV, USA) followed by lowpass whole genome sequencing on an Illumina NovaSeq™ platform.

### 2.6. Data Analysis

Copy number profile analysis was performed by Menarini Silicon Biosystems using the Control-FREEC software and ploidy levels were automatically estimated by the MSB pipeline based on the underlying copy number levels [24].

## 3. Results

Recovery rates for this workflow were evaluated using different cell lines and ranged between 19% and 33% (Appendix A, Figure A1). Applying the Menarini Silicon Biosystems protocol, established for the detection of CK-positive CTCs, OVCAR-3 cells were used to establish and optimize ERCC1- and CD11b antibody concentration and DEPArray^TM^ NxT settings for single cell detection (Appendix A, Figure A2 and Figure A3). When we applied the optimized staining protocol in patient-derived samples, we frequently detected the following three cell types:

1. Type A-cells (epithelial sCTC):
  Hoechst^pos^, ERCC1^pos^, CD45^neg^, CD11b^pos^, CK^pos^;
2. Type B-cells (potential epithelial sCTC):
  Hoechst^pos^, ERCC1^pos^, CD45^neg^, CD11b^pos^, CK^neg^;
3. Type C-cells (potential mesenchymal sCTC):
  Hoechst^pos^, ERCC1^pos^, CD45^neg^, CD11b^neg^, CK^neg^.

**Figure 2:**
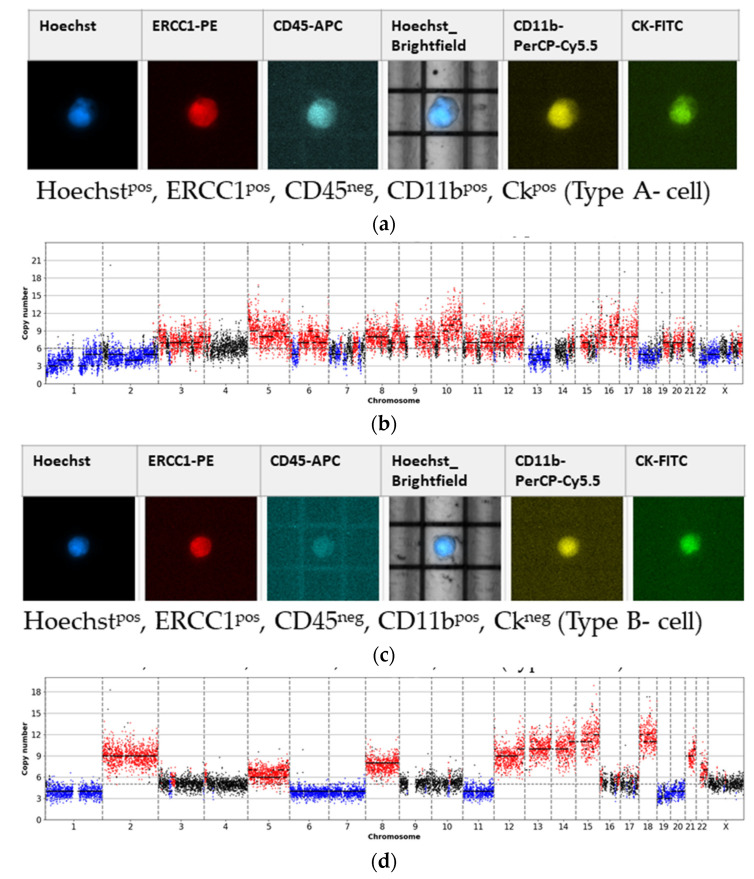

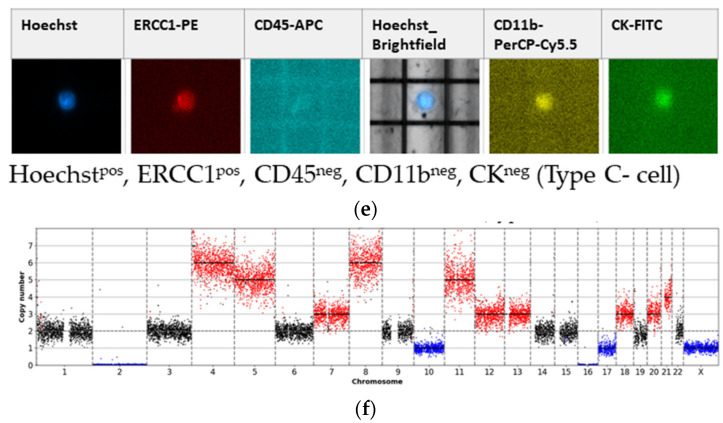
DEPArray^TM^ fluorescence panel from different single cell types and matched CN profiles. Ploidy levels were automatically approximated based on the underlying copy number levels, amplifications (red) and deletions (blue) are based on the estimated ploidy. (**a**) Type A-cell, Hoechst^pos^, ERCC1^pos^, CD45^neg^, CD11b^pos^, CK^pos^; patient 10. (**b**) Matched CN profile of Type A-cell (**a**) with a ploidy of 6. (**c**) Type B-cell, Hoechst^pos^, ERCC1^pos^, CD45^neg^, CD11b^pos^, CK^neg^; patient 11. (**d**) Matched CN profile of Type B-cell (**c**) with a ploidy of 5. (**e**) Type C-cell, Hoechst^pos^, ERCC1^pos^, CD45^neg^, CD11b^neg^, CK^neg^; patient 12. (**f**) Matched CN profile of Type C-cell (**e**) with a ploidy of 2.

Type A-cells (Figure 2a) characterized by a combined positive staining for Hoechst, ERCC1, CD11b and CK, were classified as an epithelial-like CTC, due to the CK positivity. CK-positivity was frequently linked to a CD11b-positivity, thereby, suggesting CD11b as a non-mesenchymal marker, which could extend the detection spectrum of epithelial-like CTCs in the CK^neg^ cell population (Type B-cells; Figure 2c). Type C- cells (Figure 2e), solely positive for Hoechst and ERCC1, could identify potential mesenchymal CTCs. We estimated the CTC burden in each cell-subclass by further downstream analysis of whole genome CNV.

In 11 out of 13 patient samples, 25 Type A-cells were detected whereas Type B- and C-cells were identified in every patient in higher numbers and therefore, we waived an evaluation of actual cell counts in the two latter groups. Intending to gain information about the different cell types, we analyzed cells of every cell type in every patient. Due to low occurrence and/or poor DNA quality after WGA, Type A-cells could be analyzed in nine patients while, Type B and Type C- cells in 11 and 12 patients, respectively.

**Figure 3:**
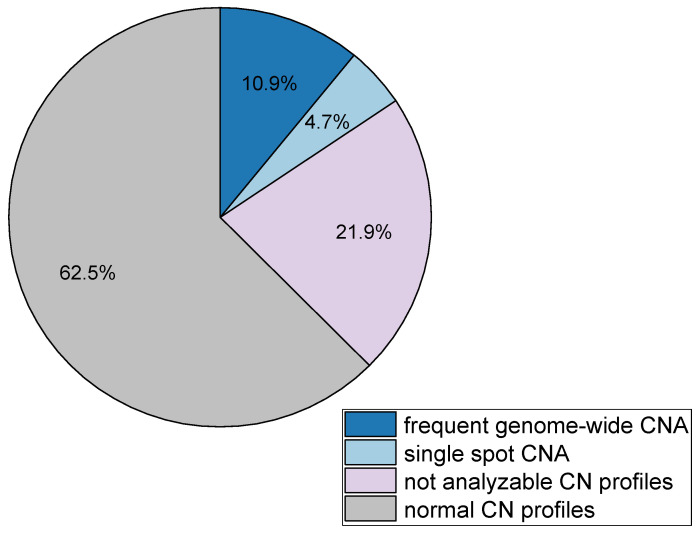
Copy number alteration frequency in all analyzed single cells.

**Figure A4:**
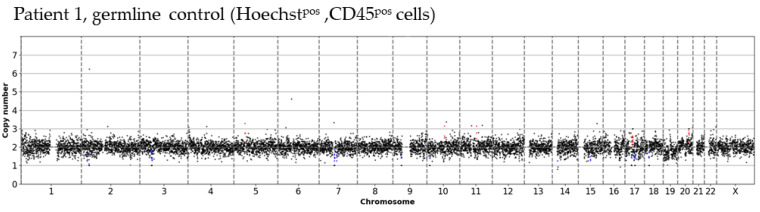

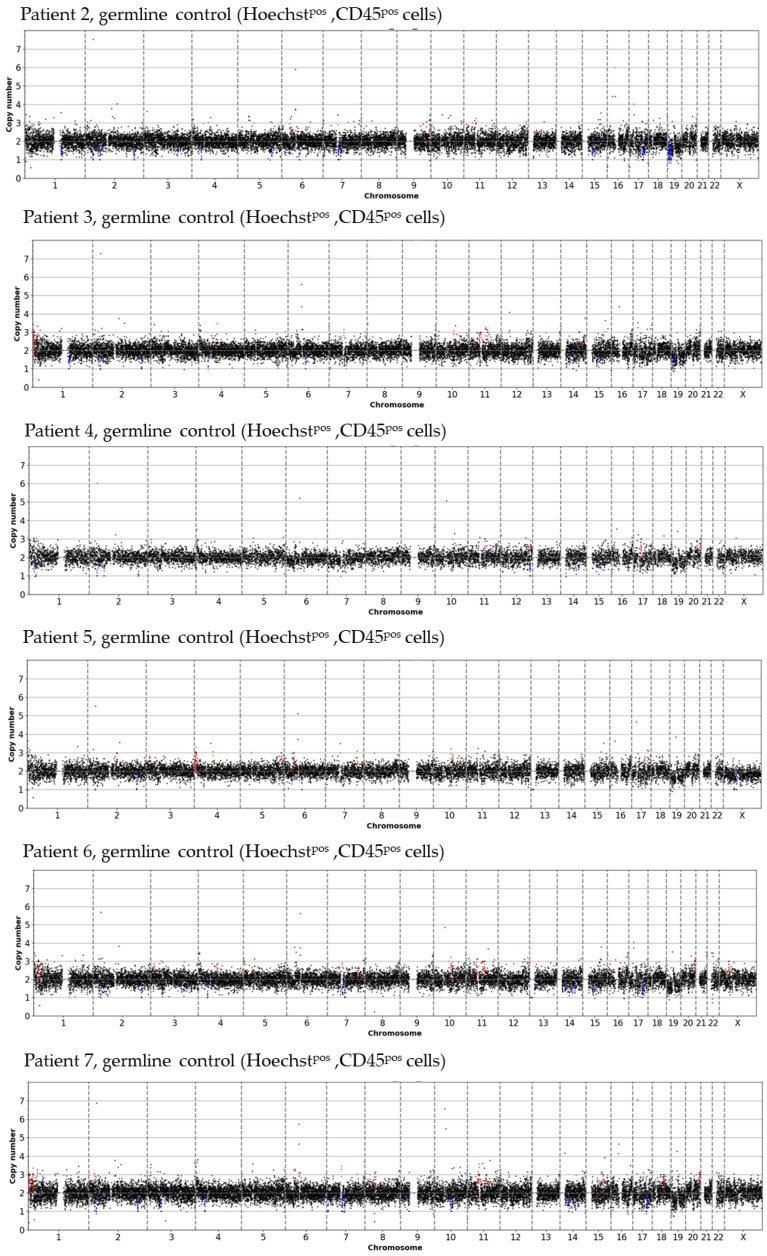

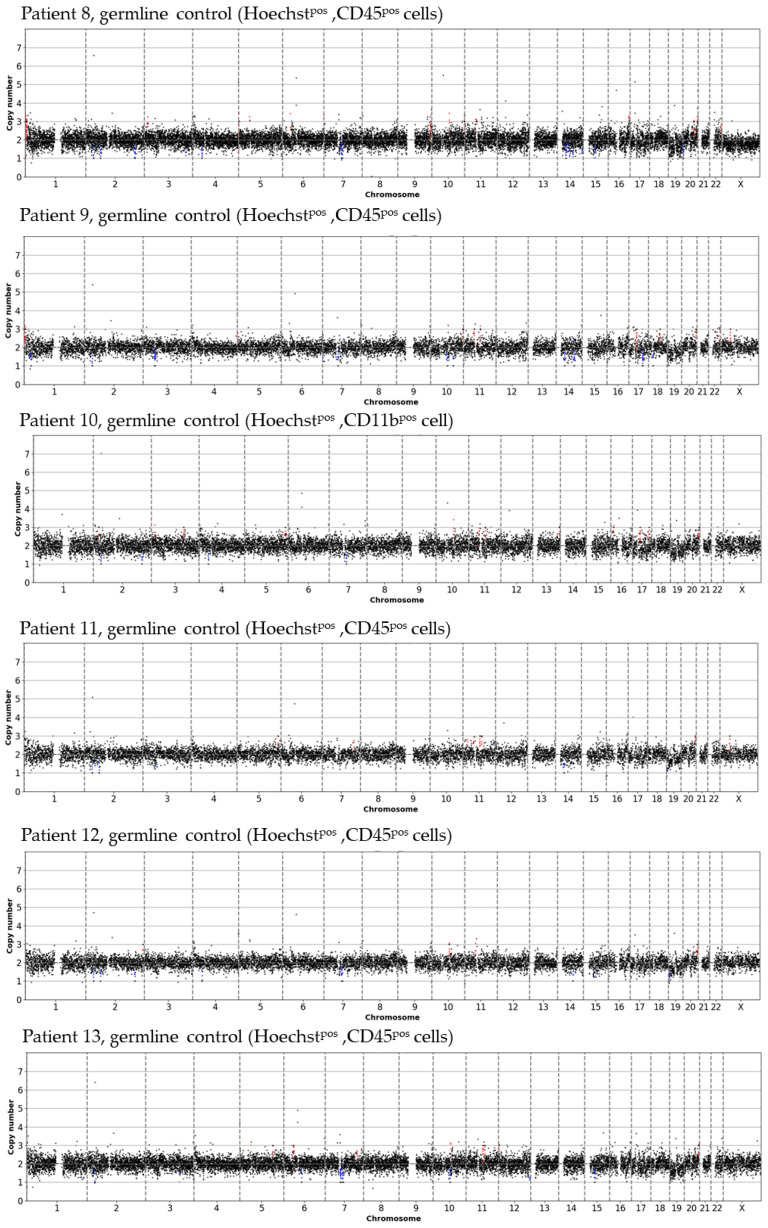
CN profiles of germline samples from all 13 patients. Cells (range 7-10) positive for CD45 and the nucleus counter staining Hoechst were isolated in every patient. For patient 10, the quality of the WGA product was not compatible with the further downstream analysis, therefore, one Hoechst^pos^ and CD11b^pos^ cell was used as a germline sample.

**Figure A5:**
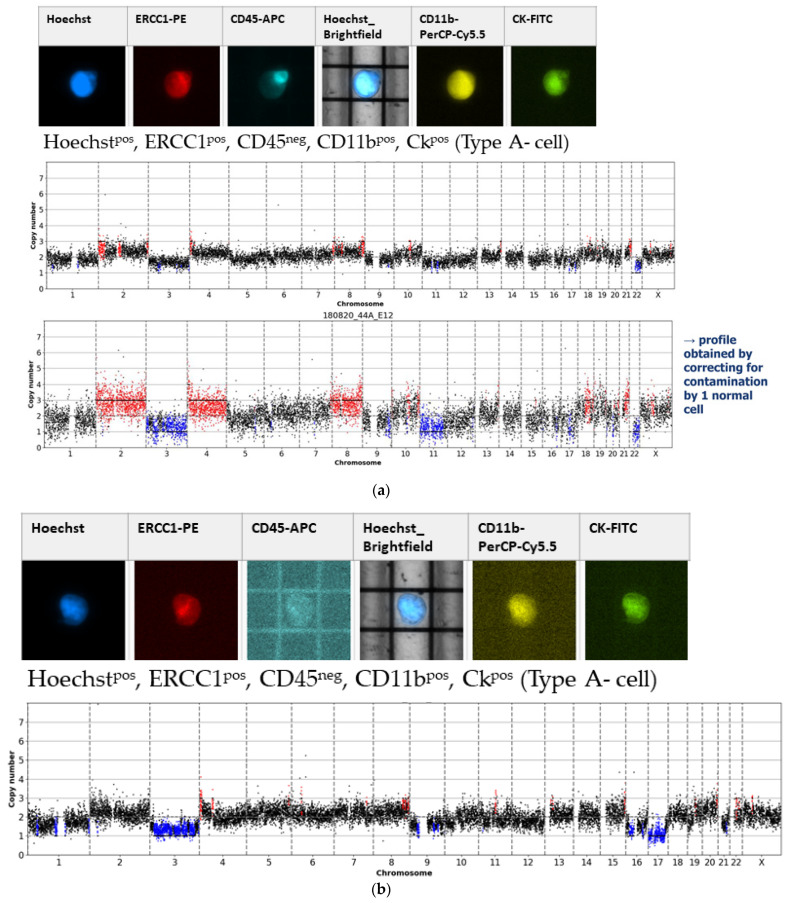

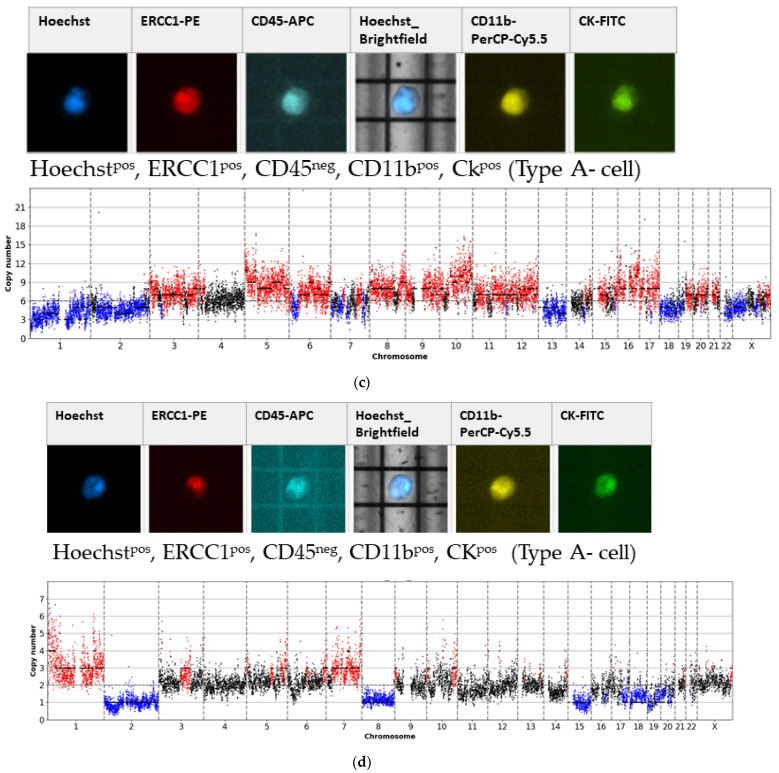

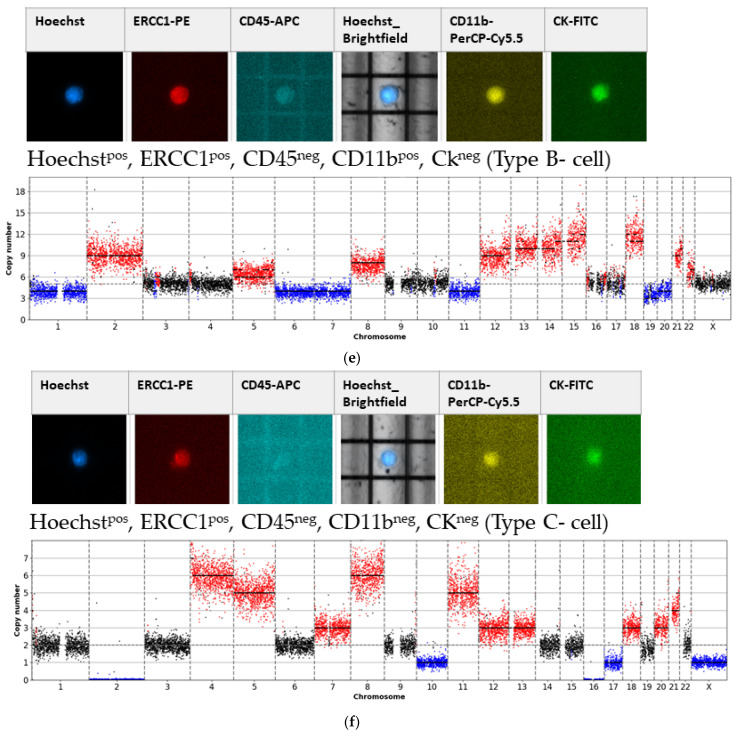

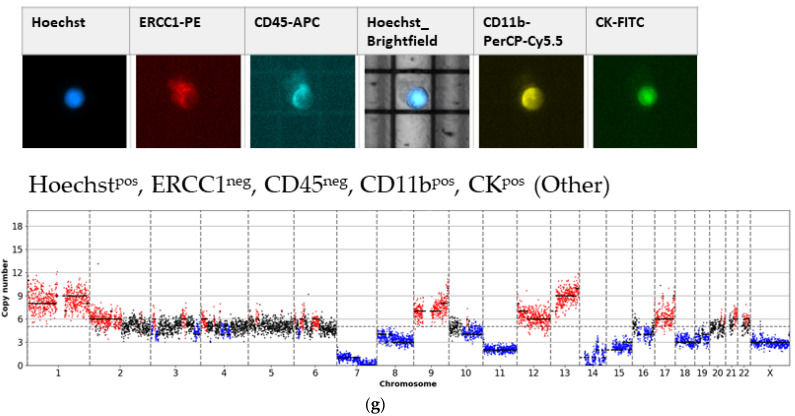
DEPArray^TM^ fluorescence panel of single cells with matched CN profiles showing genome wide CNAs. (**a**) Type A-cell (Hoechst^pos^, ERCC1^pos^, CD45^neg^, CD11b^pos^, CK^pos^) with matched CN profile and a ploidy of 2 from patient 8. (**b**) Type A-cell (Hoechst^pos^, ERCC1^pos^, CD45^neg^, CD11b^pos^, CK^pos^) with matched CN profile and a ploidy of 2 from patient 9. (**c**) Type A-cell (Hoechst^pos^, ERCC1^pos^, CD45^neg^, CD11b^pos^, CK^pos^) with matched CN profile and a ploidy of 6 from patient 10. (**d**) Type A-cell (Hoechst^pos^, ERCC1^pos^, CD45^neg^, CD11b^pos^, CK^pos^) with matched CN profile with a ploidy of 2 from Patient 11. (**e**) Type B-cell (Hoechst^pos^, ERCC1^pos^, CD45^neg^, CD11b^pos^, CK^neg^) with matched CN profile with a ploidy of 5 from Patient 11. (**f**) Type C-cell (Hoechst^pos^, ERCC1^pos^, CD45^neg^, CD11b^neg^, CK^neg^) with matched CN profile with a ploidy of 2 from Patient 12. (**g**) Other cell type (Hoechst^pos^, ERCC1^neg^, CD45^neg^, CD11b^pos^, CK^pos^) with matched CN profile with a ploidy of 5 from patient 11.

**Figure A6:**
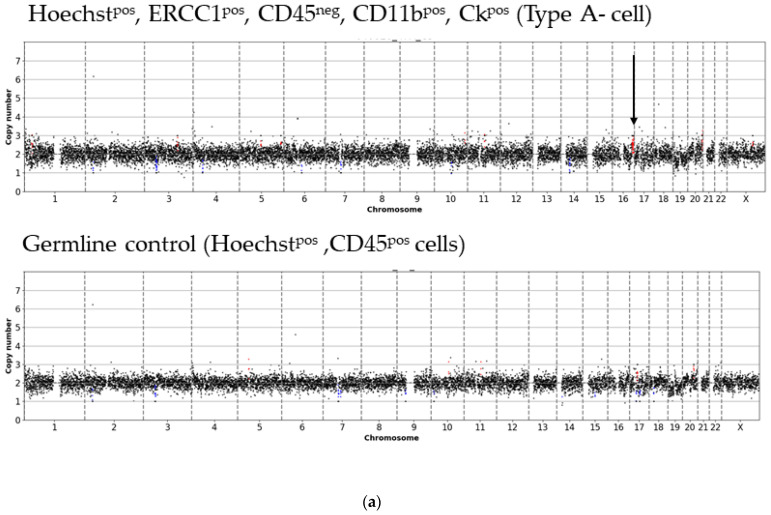

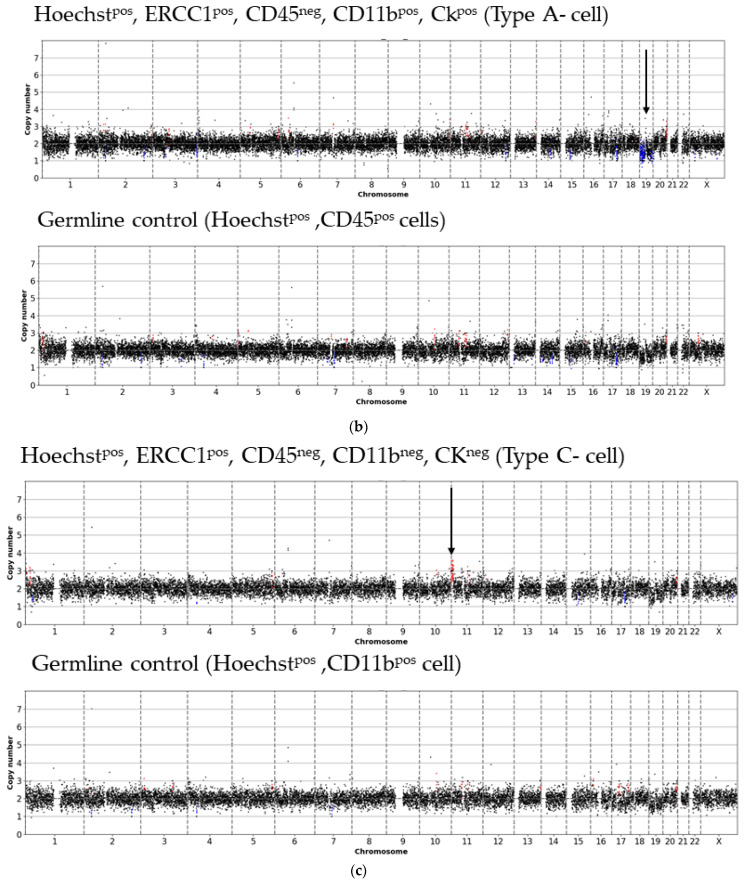
Three CN profiles with single spot CNAs from three different patients. (**a**) Patient 1 with a single spot CNA at chromosome 16 in a Type A- cell, compared to the matched germline CN profile with no aberrations. (**b**) Patient 6 with a single spot CNA at chromosome 19 in a Type A- cell, compared to the matched germline CN profile with no aberrations. (**c**) Patient 10 with a single spot CNA at chromosome 11 in a Type C- cell, compared to the matched germline CN profile with no aberrations. Due to low DNA quality of the CD45^pos^ cells, a Hoechst^pos^ and CD11b^pos^ cell was used as germline control.

In total, we estimated copy number profiles of 77 samples, 64 single Type A/B/C cells (range 5–7 in every patient) and 13 samples of CD45^pos^cells processed and isolated similarly in every patient (Appendix A, Figure A4). Single cell CN profiles were compared to the matched germline CN profiles (CD45^pos^ cells), to estimate their aberrant character. Frequent and genome-wide CNAs (shown in Appendix A, Figure A5) were used to confirm sCTC attributes, whereas the cells with single spot CNAs were not included in the evaluation of sCTCs, due to unknown significance of those aberrations. Cells with single spot CNAs and their matched germline CN profiles can be found in Appendix A (Figure A6). 10.9% (seven cells) of all analyzed cells showed genome-wide CNAs, whereas 4.7% (three cells) showed only single spot CN changes with unclear significance, 62.5% (40 cells) presented with normal CN profiles, while 21.9% (14 cells) were not analyzable due to poor profile quality (Figure 3).

### 3.1. Detected Cell Types and Their Copy Number Alterations

**Figure 4:**
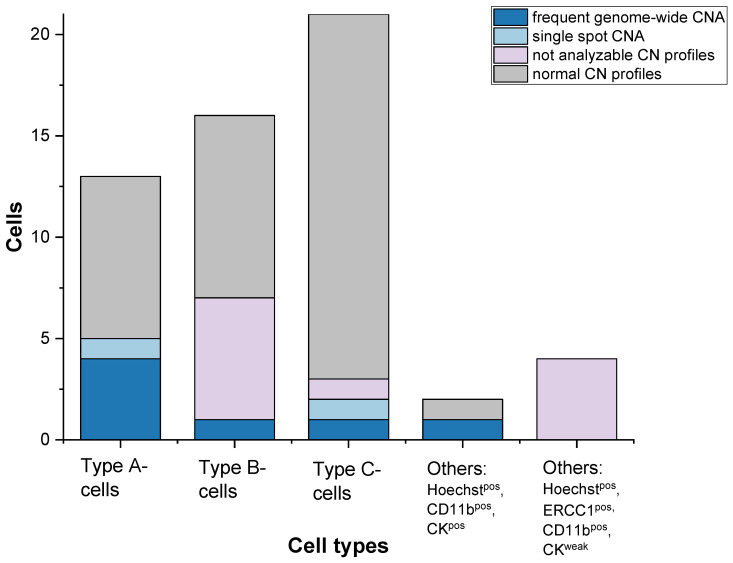
Copy number alteration frequency in different single cell types.

In 30.8% of Type A- cells (Figure 4), their aberrant character was underlined through frequent genome-wide CNA with one representative profile illustrated in Figure 2b. In contrast, in Type B-cells as well as in Type C-cells, highly altered CN were only detected in 6.25% and 4.76% of sCTCs, respectively (Figure 4). Representative images are demonstrated in Figure 2d,f. Cells that were not analyzable were more frequently found among Type B-cells (37.5%) as Type C-cells (4.76%) (Figure 4). We also analyzed, two cells positive for Hoechst, CD11b and CK, solely occurring in Patient 11. Sequencing revealed one cell with a highly aberrant character and a ploidy of five, whereas the other cell presented with a normal CN profile. Notably, every analyzed cell, positive for Hoechst, CD11b, ERCC1 and only a weak positivity for CK was not analyzable due to poor CN profile quality (Figure 4).

### 3.2. Inter- and Intra-Patient Heterogeneity

In total, we identified seven cells (10.9%) from five patients (38.5%) harboring an altered CN profile, mainly Type A-cells (80%). Those cells, present with very heterogeneous alteration profiles and differences in ploidy (Appendix A, Figure A4), reflecting the heterogeneity of sCTC across patients. 57.1% (*n* = 4) aberrant single cells presented CNAs in chromosome segments, known to be frequently altered in HGSOC primary tumor tissue, as chromosome segments RB1 (13q14.2) and CDK2AP1 (12q24.31), genes affecting cell cycle progression and KRAS (12p12.1), a gene affecting proliferation and survival. The other 42.9% (*n* = 3) cells commonly presented alterations in NF1 (17q11.2) another gene of the PI3K/KRAS signaling cascade, affecting proliferation and survival.

**Figure 5:**
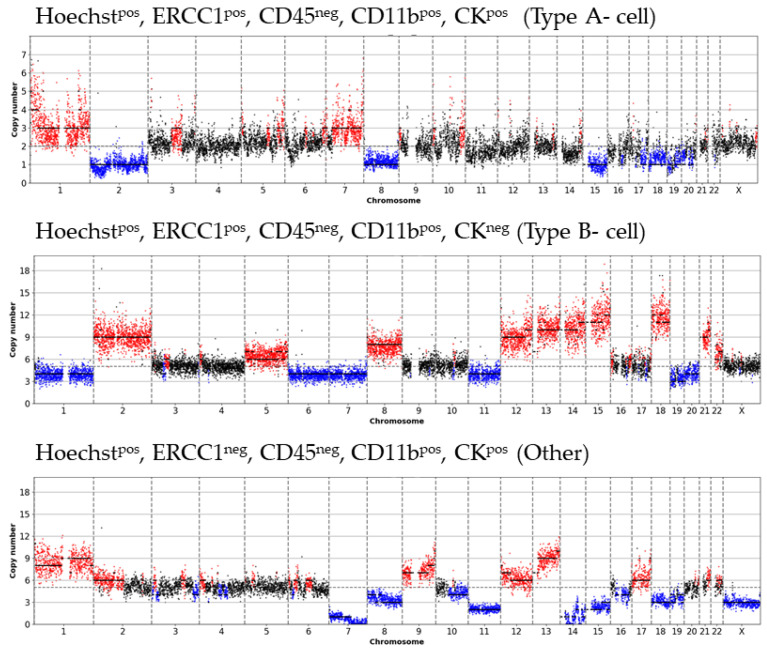
CN profiles of three phenotypic differing sCTCs (patient 11) underlining inta-patient CTC heterogeneity. (1) CN profile of one Type A- cell, Hoechst^pos^, ERCC1^pos^, CD45^neg^, CD11b^pos^, CK^pos^, (2) CN profile of one Type B- cell, Hoechst^pos^, ERCC1^pos^, CD45^neg^, CD11b^pos^, CK^neg^ and (3) CN profile of one un-defined cell, Hoechst^pos^, ERCC1^neg^, CD45^neg^, CD11b^pos^, CK^pos^ in the same patient.

For Patient 11, we obtained informative and clearly altered CN profiles from three cells: one Type A-, one Type B- cell, as well as one undefined cell (Figure 5). Notably, the three profiles of these cells differed greatly, suggesting a high level of intra-patient genomic sCTC heterogeneity.

## 4. Discussion

Epithelial-mesenchymal transition of tumor cells entering the blood stream challenges CTC detection in HGSOC. CTCs lose, at least to some extent, their original epithelial characteristics [25] and gain mesenchymal features, resulting in a multiplicity of intermediary phenotypes [13]. For the identification of CTCs with such intermediary phenotypes, we introduced a workflow based on enrichment of CTCs by negative selection followed by identification and isolation of CTCs using a novel immunofluorescence marker combination. Isolated single cells were further processed using protocols for WGA and NGS based CNA analysis, previously demonstrated to be reproducible [21,22,23]. We were able to define an aberrant single cell character by the comparison of these cells with matched germline CN profiles of our patient cohort and focused on investigating three frequently occurring cell-types, namely Type A-cells (Hoechst^pos^, ERCC1^pos^, CD45^neg^, CD11b^pos^, CK^pos^), Type B-cells (Hoechst^pos^, ERCC1^pos^, CD45^neg^, CD11b^pos^, CK^neg^) and Type C-cells (Hoechst^pos^, ERCC1^pos^, CD45^neg^, CD11b^neg^, CK^neg^). We present a methodology to identify and analyze sCTCs, whereby CK positivity was most valuable for CTC detection. However, within different marker combinations in the CK^neg^ cell population, we were able to isolate additional malignant cells, genetically distinct from the CK^pos^ cells.

In our study, cells positive for CK, here referred to Type-A cells, were most likely to be confirmed as sCTCs (30.8%) by frequent genome-wide CNA, reflecting the current literature, where CTCs were more likely to be detected through epithelial features, even though CTC detection rates relying on those features are very low in OC [11,12]. Interestingly, only a small amount of these identified Type A-cells had an altered CN profile, highlighting the importance of subsequent molecular analysis after implementing new image-based identification markers, notably CK-positive cells could also be circulating endothelial cells.

CNAs affecting common signaling pathways, previously described in primary HGSOC tissue [2,3,4], were also altered in our patients’ sCTC. Even though the detection rates of sCTC harboring altered genomes were low (10.9%), we were able to display sCTC heterogeneity across patients on the genomic level between phenotypic similar cells (Type- A cells). In line with another single cell study in a different cancer type [26], we were also able to display phenotypic sCTC heterogeneity within one patient, which was underlined by their differing CN profiles. Those diverse sCTCs within the same patient might build the foundation for evolution towards sCTC with survival advantages.

Moreover, we were able to describe the occurrence of CTCs expressing CD11b, as it has been previously described in primary HGSOC cancer tissue [5]. Genomically altered (frequent genome-wide CNA) Type A- cells, one Type B- cell, as well as one cell of a not further specified type showed detectable CD11b expression. Among other functionalities, CD11b activation in neutrophils delays apoptosis, which might represent a survival benefit for CTCs expressing this molecule [27]. Gaining immune features (e.g., CD45, CD14, CD16) by cancer cells was recently described by the spontaneous fusion of tumor cells with macrophages to circulating hybrid cells [28].

We also applied an additional ERCC1 staining to potentially identify mesenchymal-like CTCs solely positive for ERCC1 and the nucleus counter staining Hoechst, here referred to as Type C-Cells. The frequency of Type C-cells was too high to expect that all these cells have a malignant character and indeed, CNA analysis of these cells mostly showed normal CN profiles, therefore, they might refer to immune cells. In this regard, although performed in a different experimental setup, CTC “bulk” studies support our findings in the way that ERCC1 expression was most valuable when detected in combination with established CTC identification markers [9]. Interestingly, among all analyzed Type C-cells, only one detected cell showed frequent genome-wide CNAs, probably a mesenchymal-like sCTC. In view of our previous publications, where we identified a quite high proportion of CTCs in EMT among the whole CTC population, we expected a higher number of mesenchymal-like CTCs. Compared to the epithelial CTC identification marker CK, the regular microscopically- detectable ERCC1 expression in peripheral blood mononuclear cells is not suitable for an extended CTC detection in HGSOC. Furthermore, CD11b expression in combination with ERCC1 expression did not substantially extend the CTC detection spectrum in our patient cohort. Therefore, the significance of mesenchymal-like sCTC in our current study cohort remains unclear. Further studies are needed to investigate CD11b expression on CTCs in combination with other, probably more specific markers, to clarify whether CD11b expression can identify epithelial-like CTCs in the absence of CK expression.

In previous studies we showed that CTCs in EMT were shown to expand under therapy [14] and since this study is planning multiple blood sampling during the treatment course, to investigate sCTC on CNA- and mutational level, we might be able to address this question in the future. The number of patients of the present proof of principle study is too small and should be seen as hypothesis generating.

In general, this study presents a complex experimental setup, failing to expand CTC detection apart from well-established epithelial identification markers like CK. Nevertheless, we were able to detect and analyze HGSOC CTCs at a single cell resolution by frequent genome-wide CNAs. Further studies should investigate identification marker combinations to broaden the phenotypic CTC detection, implementing epithelial as well as mesenchymal markers. Our study presents a valuable example for the importance of genome analysis of sCTCs after identification by new additive identification markers, before implementing them in solely image-based identification methods. Moreover, our identification of sCTCs by genome-wide CNA cannot identify sCTCs that could eventually have a high mutational burden without those frequent CNA. There is a need for a definition, implementing to what extent of a copy number aberration is able to identify a CTC. It is known that hematologic cells can also present CNA [29] and a definition will help building a foundation for comparable sCTC studies. In general, this comprehensive workflow is associated with a rather high cell loss, so that the influence on the remaining cell populations remains unclear. In addition, detected CTCs might be biased towards the presence of robust and non-apoptotic sCTCs. Cells which entered a pre-apoptotic state are consequently not analyzable due to apoptosis related DNA fragmentation. This could also explain why Type A-cells presenting with only a weak CK staining were not analyzable for CNA, probably these cells represent pre-apoptotic cells.

## 5. Conclusions

We were able to identify sCTCs through a Hoechst^pos^, ERCC1^pos^, CD45^neg^, CD11b^pos^, CK^pos^ (Type A-cells) staining and subsequent whole genome copy number profiling confirmed their aberrant character in different HGSOC patients. We were able to display inter-patient heterogeneity of phenotypic similar sCTCs as well as intra-patient heterogeneity of phenotypic different sCTCs. Apart from epithelial characteristics, other marker combinations failed to detect CTCs in a respectable manner. In conclusion, EMT is one of the major challenges to reliably detect CTCs in HGSOC and further studies are needed, to improve the CTC detection rates, to finally enable one to insight tumor evolution at a sCTC resolution.

## Figures and Tables

**Table 1:** Clinical characteristics of high-grade serous ovarian cancer patients.

Patients	Age	FIGO	BRCA Mutation	Neoadjuvant ^1^	Therapy	R0 Resection ^2^
Patient 1	59	IVa	No	No	Carbo/Pac + Bev ^3^	Yes
Patient 2	77	IIIC	n.a.	No	Carbo/Pac ^4^	Yes
Patient 3	70	IIC	No	Yes	Carbo/Pac	Yes
Patient 4	61	IC	n.a.	No	Carbo/Pac	Yes
Patient 5	65	IIIB	Yes	Yes	Carbo/Pac	No
Patient 6	57	IA	n.a.	No	Carbo mono ^5^	Yes
Patient 7	60	IIIC	n.a.	No	Carbo/Pac + Bev	n.a.
Patient 8	72	IIIB	n.a.	Yes	Carbo/Pac + Bev (DUO-O Study ^6^)	No
Patient 9	71	IVb	n.a.	No	Carbo/Pac + Bev	Yes
Patient 10	52	IVb	No	No	Carbo/Pac	Yes
Patient 11	60	IVb	n.a.	No	Carbo/Pac + Bev	Yes
Patient 12	79	IIIC	No	No	Carbo/Pac + Nira ^7^	Yes
Patient 13	62	IIB	No	No	Carbo/Pac	Yes

^1^ Three therapy cycles administered bevor surgery and three cycles administered after surgery. ^2^ Macroscopically complete tumor resection. ^3^ Six Cycles Carboplatin and Paclitaxel, additionally Bevacizumab every three Weeks. ^4^ Six Cycles Carboplatin and Paclitaxel. ^5^ Six Cycles Carboplatin. ^6^ Six Cycles Carboplatin and Paclitaxel, Bevacizumab every three Weeks and Durvalumab/Placebo + Olaparib/Placebo. ^7^ Six Cycles Carboplatin and Paclitaxel, Niraparib maintenance.

## Data Availability

Data presented in this study are available in this article. Further data can be obtained from the author.

## Appendix A

The recovery rates for negative immunomagnetic enrichment followed by single cell sorting with the DEPArray^TM^ were estimated using different tumor cell lines. An approximate number of 50 breast cancer cells (SK-BR-3, MCF-7) or OC cells (OVCAR-3) were added to 10 mL EDTA healthy donor blood (*n* = 3, for every cell line) and the recovery rates were estimated based on Hoechst^pos^ and CK^pos^ cells detected with the DEPArray^TM^ resulting in 33% for SK-BR-3, 24% for MCF-7 and 19% for OVCAR-3 cells, respectively.

OVCAR-3 cells were further used to optimize ERCC1- and CD11b antibody concentration for staining and DEPArray^TM^ NxT settings for single cell detection. For the final decision of each antibody concentration, two independent experiments were performed using blood from different healthy donor samples (Figure A2 and Figure A3). CK-positive staining was added to confirm the spiked OVCAR-3 cells. For involving ERCC1- staining, an Alexa Fluor^®^ 488-conjugated CK antibody (C-11, ab187773, Abcam, Cambridge, UK) was used for 20 min at 2–8 °C, dilution 1:9, while for the tests involving CD11b- staining, a PE-conjugated version of the same CK antibody (C-11, ab52460, Abcam, Cambridge, UK) for 20 min at 2–8 °C, dilution 1:9 was used. Hoechst, CK- and CD45 antibody staining was performed according to the instruction manual from Menarini Silicon Biosystems, with minor changes described in materials and methods Section 2.4. Immunofluorescence staining and single cell recovery with DEPArray™ NxT.
